# Complete Genome Sequences of *Campylobacter jejuni* Strains RM3196 (233.94) and RM3197 (308.95) Isolated from Patients with Guillain-Barré Syndrome

**DOI:** 10.1128/genomeA.01312-15

**Published:** 2015-11-05

**Authors:** Craig T. Parker, Steven Huynh, Astrid P. Heikema, Kerry K. Cooper, William G. Miller

**Affiliations:** a Produce Safety and Microbiology Research Unit, Agricultural Research Service, U.S. Department of Agriculture, Albany, California, USA; b Department of Medical Microbiology and Infectious Diseases, Erasmus MC, University Medical Center, Rotterdam, The Netherlands; c Department of Biology, California State University Northridge, Northridge, California, USA

## Abstract

Infections with *Campylobacter jejuni* subsp. *jejuni* are a leading cause of foodborne gastroenteritis and the most prevalent infection preceding Guillain-Barré syndrome (GBS). This study describes the genomes of *C. jejuni* subsp. *jejuni* HS:41 strains RM3196 (233.94) and RM3197 (308.95) that were isolated from patients with GBS in Cape Town, South Africa.

## GENOME ANNOUNCEMENT

The majority of *Campylobacter jejuni* subsp. *jejuni* infections result in an acute self-limited gastrointestinal illness; however, in a small number of patients, *C. jejuni* subsp. *jejuni* infection is followed by the development of the autoimmune neuropathy Guillain-Barré syndrome (GBS) (1). GBS can be triggered by sialylated lipooligosaccharides (LOS) on the cell surface of *C. jejuni* that exhibit molecular similarity with gangliosides on human peripheral nerves (2–4). In *C. jejuni* subsp. *jejuni* strains isolated from stool samples from patients with GBS in Cape Town, South Africa, the Penner serotype HS:41 was overrepresented (5, 6). Previously, we observed that 13 distinct clinical *C. jejuni* subsp. *jejuni* HS:41 strains from South Africa were indistinguishable by microarray-based genomic indexing (7). To further explore the genomic similarities between these GBS-related strains, we report the genomic sequences of two *C. jejuni* subsp. *jejuni* strains, RM3196 (233.94) and RM3197 (308.95), which were isolated from patients with GBS in 1994 and 1995, respectively.

Genome sequencing was performed on an Illumina MiSeq desktop sequencer using shotgun library reads. A total of 2,188,526 (RM3196) and 2,153,596 (RM3197) reads, with an average read length of 300 nucleotides (nt), were assembled *de novo* using the Roche Newbler assembler (version 2.3) and resulted in 100 total contigs (>100 bp) and 40 large contigs (5 to 77 kb) for each strain. Reference assemblies for each strain against the *C. jejuni* NCTC 11168 genome were performed within the Geneious software (version 8.1). The *de novo* large contigs and the contigs derived from the reference assembly were used to create a draft scaffold. The scaffold gaps were filled using the small-repeat *de novo* contigs and the Perl script Contig_extender3 (8). The final genomic sequences had coverages of 325× (RM3196) and 322× (RM3197). Variations of the homopolymeric GC tracts were characterized using the high-depth MiSeq reads within Geneious.

Protein-, rRNA-, and tRNA-coding genes were identified using GLIMMER3 (9) within Geneious, RNAmmer (version 1.2) (10), and tRNAscan-SE (version 1.21) (11), respectively. The genomes were annotated based on those of the *C. jejuni* strains NCTC 11168 and MTVDSCj20 (accession numbers AL111168.1 and CP008787.1, respectively). Additional annotation was performed using Geneious, identification of Pfam domains (version 26.0) (12), and BLASTP comparisons to proteins in the NCBI nonredundant database.

The complete annotated genome sequences of RM3196 and RM3197 are each 1.66 Mbp and suggest a common ancestral strain. RM3196 and RM3197 contain 1,639 and 1,632 open reading frames, respectively. The RM3196 genome contains an additional 26 fragmented coding sequences (CDSs) identified as pseudogenes, while RM3197 contains 33. Eight flagellar modification genes and five capsular biosynthetic genes possess poly(G) tracts of varied length that result in either full-length coding sequences or pseudogenes. The annotations report the most prevalent form of the genes.

Other noteworthy features possessed by both strains include the class A1 LOS locus and a *C. jejuni* Mu-like prophage. Both strains have truncated versions of the *cgtA* gene within the LOS locus that is annotated as a pseudogene.

### Nucleotide sequence accession numbers.

The whole-genome sequences and annotations were deposited with GenBank, BioProject, and BioSample under the accession numbers CP012690, PRJNA283556, and SAMN03652743 for RM3196 and CP012689, PRJNA283557, and SAMN03652757 for RM3197, respectively.
